# Conduction Electrohydrodynamics with Mobile Electrodes: A Novel Actuation System for Untethered Robots

**DOI:** 10.1002/advs.201600495

**Published:** 2017-05-22

**Authors:** Vito Cacucciolo, Hiroki Shigemune, Matteo Cianchetti, Cecilia Laschi, Shingo Maeda

**Affiliations:** ^1^ The BioRobotics Institute Scuola Superiore Sant'Anna Viale Rinaldo Piaggio 34 Pontedera (Pisa) 56025 Italy; ^2^ Department of Applied Physics Graduate School of Science and Engineering Waseda University 3‐4‐1 Okubo Shinjuku‐ku Tokyo 169‐8555 Japan; ^3^ The BioRobotics Institute Scuola Superiore Sant'Anna Viale Rinaldo Piaggio 34 Pontedera (Pisa) 56025 Italy; ^4^ Department of Engineering Science and Mechanics Shibaura Institute of Technology 3‐7‐5 Toyosu Koto‐ku Tokyo 135‐8548 Japan

**Keywords:** electrohydrodynamics, mobile robots, soft actuators, soft robotics, untethered

## Abstract

Electrohydrodynamics (EHD) refers to the direct conversion of electrical energy into mechanical energy of a fluid. Through the use of mobile electrodes, this principle is exploited in a novel fashion for designing and testing a millimeter‐scale untethered robot, which is powered harvesting the energy from an external electric field. The robot is designed as an inverted sail‐boat, with the thrust generated on the sail submerged in the liquid. The diffusion constant of the robot is experimentally computed, proving that its movement is not driven by thermal fluctuations, and then its kinematic and dynamic responses are characterized for different applied voltages. The results show the feasibility of using EHD with mobile electrodes for powering untethered robots and provide new evidences for the further development of this actuation system for both mobile robots and compliant actuators in soft robotics.

Untethered became a fundamental keyword in the mobile robotics area, representing one of the most challenging bottlenecks in the development of the field.^1^, ^2^, ^3^, ^4^ Especially looking at robotics branches featuring innovative actuation technologies, such as soft robotics, we see that untethered solutions are quite rare.^5^, ^6^, ^7^, ^8^ There are two main ways to avoid tethering: loading the power supply and transduction elements onto the robot or providing the energy from the external environment. Electrical power supplies and transduction components cannot be easily scaled down due to technological limitations, thus requiring a large size increasing in the robot.^2^ For this reason, some researchers proposed the direct use of chemical energy to obtain mechanical actuation.^1^, ^4^, ^9^ This solution, while advanced and attractive, still presents drawbacks that limit its applicability, such as the difficult interfacing with digital control. Harvesting the energy from the environment can be an effective alternative way to activate a miniaturized robot. In this framework, magnetic field actuation is one of the most common solutions, however usually it requires the robot to be at microscale while the magnets at macroscale.^10^ This scale limitation follows the fact that the area of activation depends on the strength of the magnetic field, so higher current and more coils are required to activate a wider area. Miyashita et al. developed an untethered robot activated by magnetic field at millimeter scale, using high currents (≈10 A) and powers (≈100 W) while their operational area is only a small portion of the whole system.^11^ An alternative solution consists in the use of electrohydrodynamic (EHD) forces. EHD refers to the direct conversion of electrical energy into mechanical energy of a fluid through the interaction between the electric and the flow fields.^12^, ^13^, ^14^ The main application of EHD in the literature is in the development of simple pumping devices for miniaturized cooling systems, proving its feasibility and robustness.^15^, ^16^, ^17^ Chang et al. in 2007 proposed to use the reaction body forces exerted by the fluid on the electrodes to employ EHD as a propulsion method for miniature diodes.^18^ They applied an AC medium‐intensity electric field (≈10^4^ V m^−1^) to a liquid with relatively high conductivity having particle diodes suspended on its free surface. The current is rectified by the diodes, resulting in local DC fields that produced the EHD forces by electro‐osmosis, pushing the fluid and the diodes in opposite directions. What we propose in this work is to expand the idea of using EHD reaction forces as a propulsion method for untethered devices by exploiting a different physical mechanism called conduction EHD. In conduction EHD, Coulomb forces are the dominant ones; it applies to dielectric fluids that ionize in the proximity of the electrodes in domains called heterocharge layers (**Figure** **1**a), if the intensity of the applied DC electric field exceeds a certain threshold (≈10^5^ V m^−1^).^12^, ^14^

**Figure 1:**
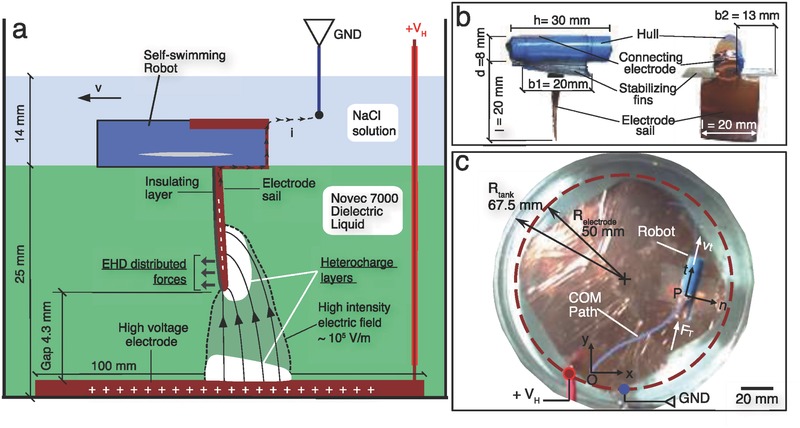
Schematics and picture of the experiment, with the illustration of the EHD phenomenon and the robot sailing in the two fluids. a) The robot is designed as an inverted sail‐boat; the high‐intensity electric field is generated between the sail and a fixed external electrode, in a volume totally immersed in the dielectric fluid; the EHD forces are exerted between the sail electrode and the ions in the heterocharge layer. In order to keep the robot untethered, we designed the electrical connection between the sail electrode and the ground by means of the conductive liquid that floats in contact with the hull, on the top of the dielectric one. b) Illustration of the robot with all the components and dimensions. c) Top view of the acceleration experiment, with the tracking of the path of the Center of Mass (COM) of the robot (see also Movie S1, Supporting Information).

The conduction EHD has been understood only recently and still a few applications have been proposed, mostly about pumping a dielectric fluid by means of two fixed electrodes.^13^, ^15^, ^19^ The use of conduction EHD with mobile electrodes, which we propose here for the first time, presents several advantages compared to other actuation methods for untethered robots: (1) it does not require bulky external hardware (e.g., coils); (2) it can work with very small electrical currents (≈10 µA) and powers ≈10 mW), allowing the use of portable power sources such as miniaturized batteries; (3) although a high intensity electric field is required (≈10^5^ Vm^−1^), it is confined in a small volume between the electrodes and it moves with them (Figure 1a), thus removing any limitation about the operational area of the robot. Additionally, EHD is widely scalable: it has been successfully applied in the MEMS area, but we show here that it can work robustly also at the millimeter scale. Respect to the electro‐osmosis proposed by Chang et al., our system does not require diodes but simple electrodes, which in our case consist of thin copper sheets with a dielectric adhesive layer on one side.^18^ Moreover, the fluids involved are dielectric, opening to the potential use of this technology in different industrial applications such as inspections in pipelines containing oils or coolants, while keeping the electrical currents at very low values. In order to test the feasibility of the system and to propose a possible application, we designed an untethered robot as an inverted sail‐boat (Figure 1a,b). The sail, i.e., the mobile electrode where the thrust is generated, is submerged in the liquid and fabricated in conductive material. The hull is on the top of it and floats on the liquid. This design produced an independent self‐sailing robot powered through the external environment. On the one hand, it represents a feasibility study for the use of conductive EHD with mobile electrodes, on the other it is an attractive application in the fields of mobile robotics and autonomous systems. In this short report our main contribution is to verify the feasibility and robustness of the use of EHD pumping as an actuation method for mobile electrodes by: (1) proposing a set‐up consisting of a millimeter scale self‐propelled robot; (2) proving that the motion of the robot was driven by EHD rather than by thermal fluctuations in the fluid; (3) measuring the response of the robot in terms of velocity; (4) computing the performance in terms of thrust produced; (5) highlighting a power‐law correlation between the delay time and maximum thrust power. We believe that this technology will find wide application in the field of untethered mobile robots, once further studies will be conducted about controlling the direction of the robot, studying the different kind of fluids that can be used and optimizing the thrust produced.

The proof that the motion of the robot, considered as a suspended particle, is a consequence of the thrust induced by EHD rather than by thermal fluctuations, can be obtained computing its diffusion constant.^20^ We conducted an experiment to measure the diffusion constant when there is no voltage applied (*V*_0_ condition) and applying a voltage of 1250 V (*V*_I_ condition). For details see Section S1 in the Supporting Information. The results are *D_V_*_0_ = 0.0272 cm^2^ s^−1^ and *D_V_*_I_ = 0.582 cm^2^ s^−1^. It is possible to observe that, when the voltage is applied, the diffusion constant increases over one order of magnitude respect to the one produced by thermal fluctuations, confirming that the motion of the robot is a consequence of the thrust induced by EHD. The second experiment had the scope of characterizing the kinematic and dynamic responses of the robot for different applied voltages. It consisted in placing the robot on one side of the tank, oriented toward the center, turning on the voltage up to the reaching of the boundary in a different point (Figure 1c; Movie S1, Supporting Information).

The trajectory described by the robot was usually curvilinear due to small asymmetries in its body and to boundary effects and convective motions of the fluid inside the tank. However, the curvatures of the trajectories were small and the robot moved mostly along its axis.

**Figure** **2**a shows the *x* and *y* time evolution of the Center of Mass (COM) of the robot actuated with different voltages. All the data were aligned such that time *t* = 0 coincides with the application of the voltage at the electrodes and the origin of the reference frame is the position occupied by the COM at that instant. Because the data were still noisy after the sampling, we computed the time derivatives of the *x* and *y* positions using the smooth derivation with noise filtering proposed by Holoborodko, using a filter length of N = 7.^21^ From the speed response, it is possible to distinguish three main regions in the behavior of the robot (Figure 2b). The first one shows latency in the EHD‐induced thrust: the robot moves with random oscillations typical of thermal fluctuations. The second one is characterized by a steep acceleration that then decreases in the third one, eventually becoming zero. In order to capture these trends and estimate the accelerations, we fitted the data with a simple trilinear model of the kind

(1) $$v(t)\;=\;\;\{\begin{array}{c} v_{0}\\ v_{0}+a_{1}\left(t-t_{1}\right)\\ v_{0}+a_{1}\left(t_{2}-t_{1}\right)+a_{2}\left(t-t_{2}\right) \end{array}\;\begin{array}{c} \mathrm{if}\;\;t\leq t_{1}\\ \mathrm{if}\;\;t_{1}<t\leq t_{2}\\ \mathrm{if}\;t\geq t_{2} \end{array}$$

where *t*_1_ is the delay time, *t*_2_ is the time threshold for the change in the acceleration, *a*_1_ and *a*_2_ are the first and second accelerations, respectively, and *v*_0_ is the average initial speed driven by thermal fluctuations. The fitting was realized through a least‐squares curve fitting with the trust‐region‐reflective algorithm, using as parameters *p* = [*v*_0_,*t*_1_, *t*_2_, *a*_1_, *a*_2_].^22^ The results are shown in **Table** **1** and in Figure 2b, where it is possible to observe that the model accurately fitted the data.

**Figure 2:**
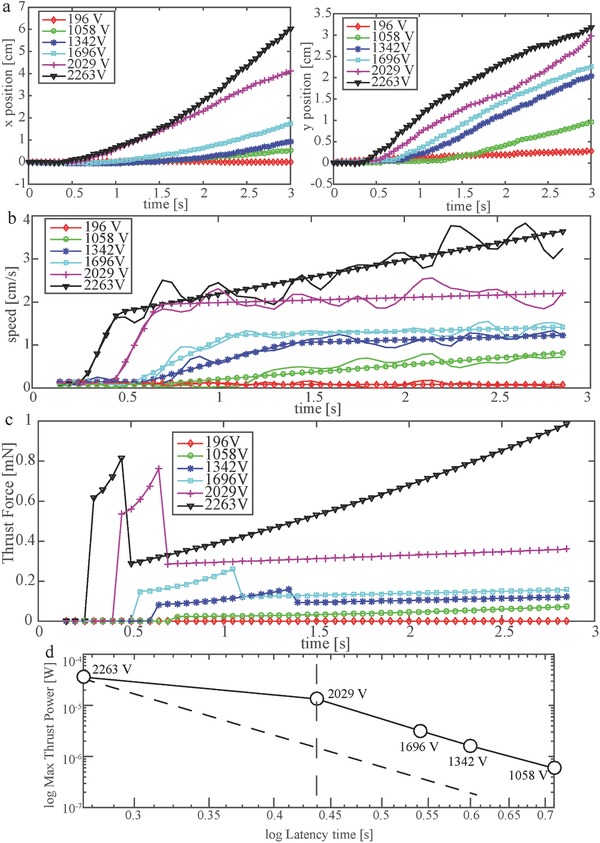
Kinematics and dynamics behavior of the robot. a) Time evolution of the *x* and *y* positions of the COM of the robot for different applied voltages. b) Tangential speed of the COM of the robot for different voltages (solid line), fitted with the trilinear model in Equation (1). c) Estimation of the thrust produced by EHD pumping on the robot, for different voltages. d) Log–log plot of the maximum thrust power versus delay time *t*_1_.

**Table 1:** Fitting parameters for the model in Equation (1)

*V*_H_ [V]	196	1058	1342	1696	2029	2263
*v*_0_ [cm s^−1^]	0.117	0.087	0.145	0.132	0.121	0.094
*t*_1_ [s]	∞	0.713	0.600	0.541	0.437	0.270
*t*_2_ [s]	∞	∞	1.378	1.051	0.673	0.458
*a*_1_ [cm s^−2^]	0	0.334	1.160	2.148	7.744	8.831
*a*_2_ [cm s^−2^]	0	0	0.119	0.105	0.118	0.791
MSE [cm s^−1^]	0.0017	0.0142	0.0088	0.0087	0.0284	0.0658

We can notice that, with *V*_H_ = 196 V, the EHD thrust was negligible and the robot moved driven by thermal fluctuations in the fluid. This was reflected in the fitting, as the accelerations *a*_1_ and *a*_2_ converged to 0, reducing to a motion at a constant average speed of *v*_0_. Such a behavior is explained by the fact that the correspondent field intensity (0.46 kV cm^−1^) is lower than the general threshold of the EHD phenomenon (≈1 kV cm^−1^). In the second voltage increment (*V*_H_ = 1058 V), the transition to the third region was never observed: the best fitting was represented by a constantly accelerated motion. From the third to the sixth increments it was instead possible to clearly identify the three regions. It is noticeable that the acceleration in the second phase *a*_1_ increased monotonically with the voltage, as shown in Table 1, while *a*_2_ was almost constant and very small up to *V*_H_ = 2263 V. The acceleration *a*_2_ was always smaller than *a*_1_. From the kinematics data, we estimated the thrust *F*_T_(*t*) generated by EHD on the robot (see Section S2 in the Supporting Information for details about the computation). Coherently with what observed in the acceleration (Figure 2b), the thrust presents latency in the beginning. The second region is dominated by inertial effects, since the robot presents here the higher acceleration, right after the appearance of the EHD phenomenon. The third region is instead characterized by the balance between drag and produced thrust (*F*_T_ ∼ *f*(*v*)). The presence of a residual acceleration, significant for *V*_H_ = 2263 V, can be explained with the transient effects characteristic of EHD, which can be peculiar in the novel case of one moving electrode proposed in this work.^23^ Finally, we investigated the correlation between the delay time *t*_1_ (Equation (1) and Table 1) and the maximum thrust power generated, for each applied voltage. The results are shown in Figure 2d. It is possible to observe that the two quantities are related by a same power law for 1058 V ≤ *V*_H_ ≤  2029 V, while there is a discontinuity for *V*_H_ ≥ 2029 V. The presence of a delay time *t*_1_ in the EHD motion has been reported in transient experiments by Tobazeon.^23^ They observed that *t*_1_ follows a power law respect to the applied voltage *t*_1_ =  *aV* ^−*k*^, where both *a* and *k* stay constant varying the voltage in a wide range (10^1^ to 10^4^ V), while the variation of the gap between the electrodes affects *a*, shifting the relationship between parallel lines in a log–log plot similar to the one in Figure 2d.^23^ The power law found in our experiments relates *t*_1_ and the maximum power rather than the applied voltage. The two phenomena are arguably strictly connected while the difference can be due to differences in the electrodes geometries, in the fluids and to the presence in our case of one mobile electrode. From the evidence presented by Tobazeon, it appears that the discontinuity shown for *V*_H_ ≥ 2029 V in our experiments can be motivated by the fact that the electrostatic force between the electrodes slightly reduced the gap between them, since the robot is not vertically constrained (see Figure 1). Therefore, we expect the point correspondent to 2263 V to belong to a power law parallel to the one shown for 1058 V ≤ *V*_H_ ≤ 2029 V, as represented by the dashed line in Figure 2d. A further investigation of the physical mechanisms observed in the proposed experiments would require a multidomain model describing: the interaction between the EHD force and the ions in the heterocharge layers; the unsteady fluid flow driven by the motion of the ions; the perturbations in the electric field and etherocharge layers due to the movement of one of the electrodes (electrode sail). Such a model requires a dedicated study and lies outside the scope of this paper. Nevertheless, the experimental results obtained in this work and the simple model proposed in (1) provide information for a further understanding of the phenomenon, which will be pursued as future work.

In this work we designed and tested an untethered sailing robot powered by conduction EHD. We demonstrated the feasibility of using conduction EHD with mobile electrodes for untethered sailing of centimeter‐scale robots, captured the dynamic response of the robot and quantified the thrust produced. This work shows a novel possible application of EHD actuation and provides data for a better understanding of this complex phenomenon. In particular, the presence of one mobile electrode in the proposed scenario introduces peculiar phenomena since the boundary conditions, which have a fundamental importance in EHD, are different from the generally studied case consisting in two fixed electrodes.^24^ One example is the possible coupling with electrostatic attraction between the electrodes, which can vary the gap as a consequence of the applied voltage, as discussed in the previous paragraph. Experiments consisting in connecting the robot to a horizontal slider at different fixed distances from the bottom electrode can contribute in quantifying this effect. On the other hand, the role of the mobility of the electrode can be investigated by moving the robot at fixed velocities and measuring the thrust produced. Although the requirements of our system, e.g., two separated layers of fluids, one conductive and the other one dielectric, high voltage, an electrode on the bottom of the tank, may seem restrictive, future improvements of this primitive set‐up will be able to remove some of these constraints and expand its applicability. One possible method for eliminating the need of the fixed bottom electrode and the conducting liquid on the top is to embed the power source and the HV converter on board and to use two electrodes both connected to the robot. The low power absorbed by EHD propulsion (≈10 mW) allows the use of miniaturized lithium batteries, while HV converters for *V*_H_ ≤ 5 kV weight ≈6 g, therefore requiring only a slight increase in the size of the robot.^25^ Furthermore, replacing mobile electrodes with compliant ones, a similar system could find application in the development of compliant actuators for soft robotics.

## Experimental Section

The experiments were conducted in a cylindrical glass tank with a diameter of 135 mm and height of 74 mm, filled with a layer of dielectric liquid topped by a layer of conductive solution (Figure 1a). The liquids that were used were Novec 7000 (1‐methoxyheptafluoropropane (C_3_F_7_OCH_3_), density ρ_N7_ = 1400 kg m^−3^, dynamic viscosity μ_N7_ = 0.450 g m^−1^ s^−1^, volume resistivity 10^8^ Ω cm) by 3 m as a dielectric and a solution of sodium chloride in water (density ρ_s_ = 1120 kg m^−3^, dynamic viscosity μ_s_ = 1.30 g m^−1^ s^−1^, resistivity 4.4 Ω cm) as conductive fluid. The Novec 7000 was chosen for the following reasons: (1) its density is higher than that of the NaCl solution and (2) the two fluids are not miscible (solubility ≤ 60 ppm by weight); (3) it is chemically stable; (4) its conductivity is very low, allowing to establish the high‐intensity electric field required by EHD and increasing the efficiency of the mechanism.^15^ On the bottom of the glass container, a copper sheet electrode with a thickness of 40 μm was glued, connected to the voltage source (+*V*_H_) through a conductive wire insulated for high voltages. As for the ground electrode, a tin wire with a diameter of 0.7 mm was inserted into the NaCl solution. In this set‐up, the positive electrode was in contact only with the dielectric fluid, while the NaCl solution only was connected to the ground (Figure 1a,c). The robot was composed by three elements (Figure 1b): (1) a hollow cylinder of polyurethane for the hull; (2) two fins made of poly(ethylene terephthalate) (PET) acting as stabilizers; (3) one submerged sail electrode, which generates the thrust, made by a square copper sheet. The extremes of the hollow cylinder were corked with hot‐melt adhesive to increase the buoyancy by sealing the internal chamber. The weight of this component was of 1.10 g, while the one of the fins 0.25 g. The electrode sail was conductive on one side only, while the other one was covered by a thin layer of dielectric glue. Its total weight was 0.20 g. All the experiments were performed using the same gap of 4.3 mm between the plane electrode on the bottom of the tank and the sail electrode on the mobile robot. This gap was chosen as a balance between obtaining enough thrust, which in EHD has been shown to depend on the gap squared and avoiding the breakdown.^13^ In all the experiments, the position of the robot was tracked through automatic tracking software (Kinovea 0.8.15) from movies acquired at 20 fps. The raw data were sampled at 5 ms with a spline interpolation.

## Conflict of Interest

The authors declare no conflict of interest.

## Supporting information

Supplementary

Supplementary

## References

[1] S. Maeda , Y. Hara , T. Sakai , R. Yoshida , S. Hashimoto , Adv. Mater. 2007, 19, 3480.

[2] M. T. Tolley , R. F. Shepherd , B. Mosadegh , K. C. Galloway , M. Wehner , M. Karpelson , R. J. Wood , G. M. Whitesides , Soft Rob. 2014, 1, 213.

[3] C. Majidi , Soft Rob. 2014, 1, 5.

[4] M. Wehner , R. L. Truby , D. J. Fitzgerald , B. Mosadegh , G. M. Whitesides , J. A. Lewis , R. J. Wood , Nature 2016, 536, 451. 27558065 10.1038/nature19100

[5] S. Kim , C. Laschi , B. Trimmer , Trends Biotechnol. 2013, 31, 287. 23582470 10.1016/j.tibtech.2013.03.002

[6] V. Cacucciolo , F. Renda , E. Poccia , C. Laschi , M. Cianchetti , Smart Mater. Struct. 2016, 25, 105020.

[7] H. Shigemune , S. Maeda , V. Cacucciolo , Y. Iwata , E. Iwase , S. Hashimoto , S. Sugano , IEEE Rob. Autom. Lett. 2017, 2, 1001.

[8] J. Shintake , S. Rosset , B. E. Schubert , D. Floreano , H. Shea , IEEE/ASME Trans. Mechatronics 2015, 20, 1997.

[9] S. Maeda , Y. Hara , R. Yoshida , S. Hashimoto , Angew. Chem., Int. Ed. 2008, 47, 6690. 10.1002/anie.200801347 18651679

[10] C. Pawashe , S. Floyd , M. Sitti , Int. J. Rob. Res. 2009, 28, 1077.

[11] S. Miyashita , S. Guitron , M. Ludersdorfer , C. R. Sung , D. Rus , IEEE Int. Conf. Robotics and Automation, IEEE, Seattle, WA, USA, 2015, p. 1490.

[12] J. R. Melcher , Continuum Electromechanics, MIT Press, Cambridge 1981.

[13] M. Pearson , J. Seyed‐Yagoobi , IEEE Trans. Dielectr. Electr. Insul. 2009, 16, 424. 10.1109/TDEI.2009.5293935 PMC2873222 20490371

[14] A. Ramos , Electrokinetics and Electrohydrodynamics in Microsystems, Springer, Vienna 2011.

[15] P. Atten , J. Seyed‐Yagoobi , IEEE Trans. Dielectr. Electr. Insul. 2003, 10, 27.

[16] S. Liang , L. Weng , S. Tan , J. Xu , X. Zhang , L. Zhang , Appl. Phys. Lett. 2007, 90, 153506.

[17] J. W. Kim , T. Suzuki , S. Yokota , K. Edamura , Sens. Actuators, A 2012, 174, 155.

[18] S. T. Chang , V. N. Paunov , D. N. Petsev , O. D. Velev , Nat. Mater. 2007, 6, 235. 17293850 10.1038/nmat1843

[19] Tasuku Sato , Yoko Yamanishi , Vito Cacucciolo , Yu Kuwajima , Hiroki Shigemune , Matteo Cianchetti , Cecilia Laschi , Shingo Maeda , Chemistry Letters 2017, DOI: 10.1246/cl.170217.

[20] A. Einstein , Ann. Phys. 1905, 17, 549.

[21] P. Holoborodko , *Smooth Noise Robust Differentiators*, http://www.holoborodko.com/pavel/numerical‐methods/numerical‐derivative/smooth‐low‐noise‐differentiators/ (accessed: March 2017).

[22] T. F. Coleman , Y. Li , SIAM J. Optim. 1996, 6, 418.

[23] R. Tobazeon , J. Electrostat. 1984, 15, 359.

[24] A. Castellanos , IEEE Trans. Electr. Insul. 1991, 26, 1201.

[25] EMCO A series, http://www.emcohighvoltage.com/proportional/aseries.php (accessed: March 2017).

