# Supplementary material for: Conduction Electrohydrodynamics with Mobile Electrodes: A Novel Actuation System for Untethered Robots
Source: Adv Sci (Weinh). 2017 May 22;4(9):1600495. doi: 10.1002/advs.201600495 (PMC5604368; doi:10.1002/advs.201600495)
Supplement: Supplementary file 1 — Supplementary [file ADVS-4-na-s001.pdf]

Copyright WILEY-VCH Verlag GmbH & Co. KGaA, 69469 Weinheim, Germany, 2017.

ADVANCED SCIENCE
Open Access

Supporting Information

for *Adv. Sci.,* DOI: 10.1002/advs.201600495

Conduction Electrohydrodynamics with Mobile Electrodes: A Novel Actuation System for Untethered Robots

Vito Cacucciolo, Hiroki Shigemune, Matteo Cianchetti, Cecilia Laschi, and Shingo Maeda**

Supporting Information

March 25, 2017

1 Diffusion Constant

Our experiment for computing the Diffusion Constant of the robot consisted in placing the specimen still in the middle of the tank, free to move passively in the liquid under thermal fluctuations for 5 seconds (V_0 condition) and then applying a voltage of 1250 V for another 5 seconds (V_I condition). We tracked the x and y positions of the Center Of Mass (COM) of the robot relative to the initial position. We repeated the experiment 20 times to obtain statistically significant data and check the repeatability and robustness of the phenomenon. With these data, we computed the diffusion constant for both the V_I and V_0 conditions as

$$D = \frac{1}{4}\frac{d\langle \bar{r}(t)^2\rangle}{dt}, \quad (1)$$

where $\langle \bar{r}(t)^2\rangle$ was obtained from a linear fitting of the mean square displacement $\langle r(t)^2\rangle$, this latter computed as $\langle r(t)^2\rangle = \Sigma_{i=1}^{n}(x(t)_i^2 + y(t)_i^2)/n$. [1]

2 Thrust

In order to compute the thrust generated by EHD on the robot submerged in the fluid, we assumed the following dynamical model

$$(m + m_a)a(t) = F_T(t) - F_D(v(t)), \quad (2)$$

where F_T is the thrust we want to estimate, m is the mass of the specimen, m_a is the added mass of the fluid, F_D is the drag force.[2]

We computed for the cylinder and electrode sail an added mass $m_a = 5.3$ g, while we neglected the contribution of the stabilizing fins (Section 2.1). As for the drag force, we took into account both the pressure and friction drags, obtaining (Section 2.2) $F_D(v(t)) = K_p * v(t)^2 + K_f * v(t)^{3/2}$, with $K_p = 0.5 * C_D * (\rho_{ws} * (\pi d^2/4) + \rho_{N7} * l^2)$, $K_f = 0.5 * 1.328 * \sqrt{\rho_{ws} * \mu_{ws}/d} * (4b_1 b_2 + \pi dh)$. Putting together the results, the thrust is

$$F_T(t) = (m + m_a)a(t) + K_p * v(t)^2 + K_f * v(t)^{3/2}. \tag{3}$$

2.1 Added Mass

As far as the computation of the added mass is concerned, we considered the specimen as constituted by a cylinder (the main body) and a square plate (the electrode sail). We instead neglected the contribution of the stabilizing wings because they exposed a very small surface to the flow. We considered the cylinder as fully submerged in the NaCl solution and the plate as fully submerged in Novec 7000 fluid. We relied on the solutions obtained through potential theory [3, 2] for the estimation of the added mass m_a. Using the fluid properties summarized in table 1 and the geometrical parameters in table 2, for a square plate moving perpendicularly to its surface we have

$$m_{a_{plate}} = 0.478\pi\rho_s l^3/4 = 4.2\text{g}. \tag{4}$$

For the cylinder, we approximated it with a parallelepiped of height h and squared base of side d and we have

$$m_{a_{cyl}} = K\rho d^2 h. \tag{5}$$

Values for K are provided in tables in the literature, and for $h/d = 3.75$ we interpolated $K = 0.47$, obtaining $m_{a_{cyl}} = 1.1$ g.[3] So the total added mass is $m_a = m_{a_{plate}} + m_{a_{cyl}} = 5.3$ g.

2.2 Drag

In order to compute the drag $F_D(v(t))$, we first of all computed the Reynolds number (Re) governing the fluid flow around the robot. We obtained two different values for the cylinder $Re_{cyl} = v * 6.9 \cdot 10^3$ and for the electrode fin

Table 1: Fluids properties

Fluid	Novec 7000	NaCl solution
concentration [M]	n/a	3.8
density [kg m^{-3}]	$\rho_{N7} = 1400$	$\rho_s = 1120$
dyn. viscosity [g m^{-1} s^{-1}]	$\mu_{N7} = 0.450$	μ_s [4] $= 1.30$
resistivity [Ohm cm]	10^8	4.4

Table 2: Geometrical parameters

Parameter	value [mm]
h	30
d	8
l	20
b_1	20
b_2	13

$Re_{plate} = v * 62.2 \cdot 10^3$, using as characteristic dimensions d for the cylinder and l for the fin. In the ranges of speeds involved in our experiment we can consider always the flow as laminar ($1 < Re < 10^4$) because $Re_{plate} \in [62, 2500]$, $Re_{cyl} \in [6.9, 276]$ for $v \in [0.1, 4]$ cm s^{-1}. In this laminar flow regime, both the friction drag F_{DF} and pressure drag F_{DP} should be taken into account. Given that both the cylinder and the plate electrode have sharp edges, according to Hoerner [5] it is possible to consider the drag coefficient approximately constant for our Re values: $C_D \approx 2$. As far as the frictional drag is concerned, we will use the model proposed by Blasius [6] for flat surfaces parallel to the flow: $C_F = 1.328 * \sqrt{Re}$. With these coefficients, the drag forces will be $F_{DP} = 0.5 * C_D \rho S v^2$ and $F_{DF} = 0.5 * C_F \rho S_w v^2$, where S is the surface perpendicular to the flow, while S_w is the one parallel to the flow. We took into account F_{DP} for the cylinder and the electrode sail and the F_{DF} for the cylinder and the lateral wings. We neglected the pressure drag for the wings (S small) and the friction drag for the electrode sail (S_w

small). With these considerations, we obtained

$$\begin{cases} F_{DP}(v(t)) = [0.5 * C_D * (\rho_{ws} * (\pi d^2/4) + \rho_{N7} * l^2] * v(t)^2 \\ F_{DF}(v(t)) = [0.5 * 1.328 * \sqrt{\rho_{ws} * \mu_{ws}/d} * (4ab + \pi dh)] * v(t)^{3/2} \\ F_D(v(t)) = F_{DP}(v(t)) + F_{DF}(v(t)) \end{cases} \quad (6)$$

References

[1] A. Einstein, *Annalen der Physik* **1905**, *17*, 549–560.

[2] R. J. Fritz, *J Eng Ind Trans ASME* **1972**, *94 Ser B*, 167–173.

[3] K. T. Patton, *Tables of Hydrodynamic Mass Factors for Translational Motion*, ASME, New York, N.Y., American S ed., **1965**.

[4] J. Kestin, H. E. Khalifa, R. J. Correia, *Journal of Physical and Chemical Reference Data* **1981**, *10*, 71–88.

[5] S. F. Hoerner, *Fluid-dynamic Drag: Practical Information on Aerodynamic Drag and Hydrodynamic Resistence*, Sighard F. Hoerner, **1965**.

[6] J. N. Newman, *Marine hydrodynamics*, MIT Press, **1977**.
